# Blood Lead Concentrations in Children and Method of Water Fluoridation in the United States, 1988–1994

**DOI:** 10.1289/ehp.8319

**Published:** 2005-08-17

**Authors:** Mark D. Macek, Thomas D. Matte, Thomas Sinks, Dolores M. Malvitz

**Affiliations:** 1 Department of Health Promotion and Policy, Baltimore College of Dental Surgery, Dental School, University of Maryland, Baltimore, Maryland, USA; 2 Division of Oral Health, National Center for Chronic Disease Prevention and Health Promotion,; 3 National Center for Environmental Health, and; 4 Agency for Toxic Substances and Disease Registry, Centers for Disease Control and Prevention, Atlanta, Georgia, USA

**Keywords:** adolescents, children, fluoridation, nutrition surveys, lead, United States

## Abstract

Some have hypothesized that community water containing sodium silicofluoride and hydrofluosilicic acid may increase blood lead (PbB) concentrations in children by leaching of lead from water conduits and by increasing absorption of lead from water. Our analysis aimed to evaluate the relation between water fluoridation method and PbB concentrations in children. We used PbB concentration data (*n* = 9,477) from the Third National Health and Nutrition Examination Survey (1988–1994) for children 1–16 years of age, merged with water fluoridation data from the 1992 Fluoridation Census. The main outcome measure was geometric mean PbB concentration, and covariates included age, sex, race/ethnicity, poverty status, urbanicity, and length of time living in residence. Geometric mean PbB concentrations for each water fluoridation method were 2.40 μg/dL (sodium silicofluoride), 2.34 μg/dL (hydrofluosilicic acid), 1.78 μg/dL (sodium fluoride), 2.24 μg/dL (natural fluoride and no fluoride), and 2.14 μg/dL (unknown/mixed status). In multiple linear and logistic regression, there was a statistical interaction between water fluoridation method and year in which dwelling was built. Controlling for covariates, water fluoridation method was significant only in the models that included dwellings built before 1946 and dwellings of unknown age. Across stratum-specific models for dwellings of known age, neither hydrofluosilicic acid nor sodium silicofluoride were associated with higher geometric mean PbB concentrations or prevalence values. Given these findings, our analyses, though not definitive, do not support concerns that silicofluorides in community water systems cause higher PbB concentrations in children. Current evidence does not provide a basis for changing water fluoridation practices, which have a clear public health benefit.

The ability of fluoride to prevent dental caries has been well documented across various populations and study conditions (Booth et al. 1992; Brunelle and Carlos 1990; Burt et al. 1986; Clark et al. 1995; Eklund et al. 1987; Gilchrist et al. 2001; Newbrun 1989; Rugg-Gunn et al. 1988). Three primary mechanisms of action have been identified (Burt and Eklund 1999): *a*) promotion of remineralization and inhibition of demineralization of early lesions; *b*) inhibition of bacterial metabolism; and *c*) reduction of enamel solubility in acid, bestowed prior to tooth eruption. In 2000, the Centers for Disease Control and Prevention (CDC) estimated that 162.1 million Americans were receiving fluoridated water, which is 57.6% of the total population and includes 65.8% of those on public water systems (Apanian et al. 2002). In the United States, several agents are used to fluoridate community water supplies, including silicofluoride compounds (sodium silicofluoride and hydrofluosilicic acid) and sodium fluoride.

The adverse health effects of lead have been described in detail. In children, elevated concentrations of lead are associated with impairment of cognitive development and adverse behavioral changes [Agency for Toxic Substances and Disease Registry (ATSDR) 1999; Johnston and Goldstein 1998]. For children age 6 years or younger, elevated blood lead (PbB) concentrations are defined as those ≥ 10 μg/dL (CDC 1991). The home environment remains an important setting for lead exposure, especially for children living in older dwellings. Heavily leaded paints were used before 1950, but lead compounds continued to be added to some paints until the Consumer Product Safety Commission (CPSC) banned the practice in 1978 (CPSC 1977). Before the 1930s, lead was used to produce pipes for drinking water systems in the United States; although copper replaced lead in pipe production after the 1930s, lead was still used as solder until the U.S. Environmental Protection Agency (EPA) banned leaded solder and pipes in 1986 (U.S. EPA 1986). As a result of the historic patterns of lead use in housing, the oldest dwellings contain more leaded paint and lead-contaminated dust (Jacobs et al. 2002), and children who live in these homes are more likely to have elevated PbB concentrations (Pirkle et al. 1998).

Two studies have reported ecologic associations between use of silicofluoride compounds in community water systems and elevated PbB concentrations among children in Massachusetts and New York (Masters and Coplan 1999; Masters et al. 2000). In the Massachusetts study (Masters and Coplan 1999), the authors stated that children who lived in communities with old housing were at increased risk for elevated PbB concentrations. In the New York study (Masters et al. 2000), the authors concluded that the highest likelihood of elevated PbB concentrations occurred when children were exposed to both water treated with silicofluorides and another risk factor known to be associated with high blood lead, such as old housing. These studies had some important limitations, however, including the lack of data on covariates at the individual level, unclear sampling methods, and use of highly skewed, untransformed PbB concentration data in analysis-of-variance models. In this analysis, we tested possible associations between water fluoridation method and PbB concentrations in U.S. children using a representative sample and addressing some of the limitations of earlier studies.

## Materials and Methods

### Study population.

PbB concentration data and covariates for children aged 1–16 years were obtained from the Third National Health and Nutrition Examination Survey (NHANES III), a cross-sectional survey of the civilian, noninstitutionalized population of the United States. NHANES III was administered by the National Center for Health Statistics (NCHS) between 1988 and 1994, with participants sampled according to a complex, multistage probability sampling method. Young children, older adults, non-Hispanic blacks, and Mexican Americans were oversampled so that population estimates for these population groups would be statistically reliable. Detailed descriptions of the NHANES III methodology have been published elsewhere (NCHS 1994, 1996).

There were 13,944 children 1–16 years of age eligible for inclusion in NHANES III, of whom 9,477 had a known PbB concentration measurement. There was no significant difference in fluoridation status between children with a known PbB concentration and those with an unknown or missing PbB concentration. The overall response rate for this analysis was 68.0%. The final sample represented 52.2 million U.S. children.

### Assignment of water fluoridation exposure.

Between 1975 and 1992, the CDC periodically collected water fluoridation status information from states and published this information in a series of monographs called the Fluoridation Census. For the 1992 Fluoridation Census, the CDC sent a printout of water fluoridation status data from the 1989 Fluoridation Census to each state. A responsible person in the health or water departments was asked to update, edit, and verify the information. Edits were made to reflect installations of new water systems, systems that had stopped fluoridation, and changes in population. In addition, states were asked to report *a*) each fluoridated water system and the communities each system served; *b*) the status of fluoridation (“adjusted” to provide optimal levels; “consecutive,” i.e., water systems that purchased fluoridated water from another system; or “natural”); *c*) the system from which water was purchased (if another system served as the primary source); *d*) the date on which fluoridation started; and *e*) the chemical used for fluoridation (if adjusted to provide optimal levels or purchased from another source). The final 1992 Fluoridation Census document represented information returned from state respondents to the CDC (1993).

Information regarding the locations from which NHANES III selected its sample participants is not made available to the public because of concerns about the confidentiality of survey results and other risks of disclosure. To create an analytic file for this analysis, NCHS used the 1992 Fluoridation Census to assign a water fluoridation method value to each child in NHANES III, based on the child’s county of residence. NCHS forwarded the analytic file to us without county-level data. NCHS maintains a copy of the combined data file and provides access to this file through the NCHS Research Data Center.

NCHS classified the water fluoridation method into one of six categories (sodium silicofluoride, hydrofluosilicic acid, sodium fluoride, natural fluoride, no fluoride, and unknown/mixed status) according to the following algorithm: *a*) If at least 90% of a NHANES III county received a single type of fluoride or no fluoride, then the county was assigned that water fluoridation method category; *b*) if < 90% of a NHANES III county received a single type of fluoride or no fluoride, or if > 10% of a NHANES III county received an unidentified type of fluoride, then the county was classified as “unknown/mixed status.”

We were unable to assign a water fluoridation method to children who were not served by a public water system, so we included these children in the “unknown/mixed status” category. Furthermore, we were unable to account for changes in the type of fluoride used by water systems over time. Given that water systems do not routinely change type of fluoride used, misclassification due to changes over time would probably not have influenced our findings.

### Blood lead measurement.

Blood was collected from individual survey participants ≥ 1 year of age via venipuncture during the phlebotomy component of NHANES III. Blood specimens were analyzed for lead at the NHANES Laboratory, Division of Environmental Health Laboratory Sciences, National Center for Environmental Health, CDC, using graphite furnace absorption spectrophotometry and previously described methods (Gunter et al. 1996).

### Covariates.

Other independent variables associated with PbB concentrations were obtained from NHANES III, including age (1–5 years, 6–16 years), sex, race/ethnicity (non-Hispanic white, non-Hispanic black, Mexican American, other), poverty status [<100% of the federal poverty level (FPL), < 100% FPL, unknown], urbanicity (urban = population ≥ 250,000 persons, suburban/rural = population < 250,000 persons), duration of residence (lifetime, less than lifetime, unknown), and year in which dwelling was built (before 1946before 1946–1973–1974 to present, unknown).

### Statistical analysis.

We used SUDAAN statistical software for personal computers (Research Triangle Institute 2000) to estimate PbB concentrations and to estimate multiple linear and logistic regression coefficients for change in PbB concentration, controlling for covariates. SUDAAN accounted for the complex sampling design of NHANES III when deriving standard errors (SEs) and confidence intervals (CIs). The α-value for statistical significance was set at 0.05 for all analyses.

Because PbB concentrations have a highly positively skewed distribution, we used log-transformed PbB concentration data in all linear regression analyses, and used antilog transformations to convert mean log PbB concentration values to geometric mean (GM) PbB concentration values and to convert regression coefficients estimating changes in mean log PbB concentration to estimated ratios of GM PbB concentrations. Estimates with a corresponding SE equivalent to ≥ 30% of the estimate were identified as statistically unreliable and should be interpreted with caution.

If silicofluoride compounds in water were truly able to leach lead from drinking water conduits and/or increase absorption of ingested lead, one would expect that sodium silicofluoride and hydrofluosilicic acid would be associated with higher PbB concentrations in older dwellings, because older dwellings are more likely to have lead pipes or copper plumbing with lead solder (Berkowitz 1995) than are newer dwellings. To evaluate this hypothesis we also tested whether the year in which the dwelling was built interacted with water fluoridation method in its association with PbB concentrations.

We used crude Wald-*F*-test statistics to assess whether bivariate linear regression associations (selected characteristics versus mean log PbB concentrations) were significant. To assess whether interaction terms should be included in the multivariable models, we assessed the statistical significance of each interaction term (in the presence of its component main effect variable) using adjusted Wald-*F* statistics. When significant interactions were found, we conducted stratified analyses to measure stratum-specific associations between water fluoridation method and mean log PbB concentrations.

For comparison, we also modeled the adjusted odds of an elevated PbB concentration for each water fluoridation method across year-during-which-dwelling-was-built strata. We used a liberal 5-μg/dL cut-off to define elevated PbB concentration because the prevalence of an elevated PbB concentration using the standard 10-μg/dL cut-off (CDC 1991) was so low (3.3%).

For the linear and logistic regression analyses, the reference category for water fluoridation method was no fluoride. To compare one stratum-specific PbB concentration to the reference category, we calculated ratios of stratum-specific GMs divided by the reference GM for the no-fluoride category. This ratio showed whether the GM for that category of water fluoridation method was higher or lower than the GM for the reference. Odds ratios (ORs) derived from logistic regression also compared PbB concentration prevalence values for one category of water fluoridation method to the reference no-fluoride category.

## Results

From the NHANES III data (Table 1), we estimate that approximately one-third of American children 1–16 years of age were lifetime residents of their current dwelling, and about one-fifth in houses built before 1946. Approximately one in four lived in a county having hydrofluosilicic acid in its community water supply, and somewhat less than one-fifth lived in a county with no fluoride in its community water supply.

Overall, the GM PbB concentration for the population was 2.19 μg/dL (Table 2). As reported in earlier analyses of NHANES III data (Brody et al. 1994; Pirkle et al. 1994), younger age, male sex, minority race/ethnicity, and poverty status were each associated with higher GM PbB concentrations in children. Our analysis also showed that duration of residence was significantly associated with GM PbB concentration (*p* < 0.01), as was year in which dwelling was built (*p* < 0.01). GM PbB concentration was not associated with urbanicity (*p* = 0.14).

Despite a nonsignificant association between water fluoridation method and GM PbB concentration at the bivariate level (*p* = 0.88), the statistical interaction between fluoridation and year in which dwelling was built was associated with PbB concentration at the multivariable level (adjusted Wald-*F* = 9.3; *p* < 0.01). Consequently, the association between fluoridation and PbB concentration is shown stratified by year in which dwelling was built (Table 3). According to the stratum-specific models, fluoridation was significantly associated with PbB concentration only for the “before 1946” (adjusted Wald-*F* = 2.8; *p* = 0.03) and “unknown” (adjusted Wald-*F* = 2.8; *p* = 0.03) strata. In the before-1946 model, however, none of the individual fluoridation categories (including the silicofluorides compounds) was significantly higher than the reference no-fluoride category. In the unknown-year model, the hydrofluosilicic acid category was significantly different than the no-fluoride category: the GM PbB concentration for hydrofluosilicic acid was 45% higher. This significant association between hydrofluosilicic acid and GM PbB concentration seen in the unknown-year stratum was not observed in the other strata. In addition, there was no trend toward increasing GM ratios for the silicofluoride categories with increasing dwelling age.

Having a statistically significant interaction term while also having no statistically significant stratum-specific associations between fluoridation and GM PbB concentration was somewhat unexpected. To further investigate the association between fluoridation and PbB concentration, we conducted multiple logistic regression analysis, stratified by dwelling age.

Overall, 14.4% of the population had a PbB concentration ≥ 5 μg/dL (compared with 3.3% for the standard 10-μg/dL cut-off). Again, water fluoridation method was significantly associated with PbB concentration only for the before-1946 (adjusted Wald-*F* = 5.0; *p* < 0.01) and unknown (adjusted Wald-*F* = 9.5; *p* < 0.01) strata (Table 4). In the before-1946 model, however, neither silicofluoride category was significantly higher than the reference no-fluoride category. In the unknown-year model, both unknown/mixed status and hydrofluosilicic acid categories were significantly higher than the no-fluoride category; however, the significant association between hydrofluosilicic acid and PbB concentration seen in the unknown-year stratum was not observed in the other strata. Consistent with the linear regression findings, there was no trend toward increasing PbB concentration ORs for the silicofluoride categories with increasing dwelling age.

## Discussion

It has been hypothesized that silicofluoride compounds might enhance lead leaching from drinking water conduits and increase lead absorption from drinking water (Masters and Coplan 1999; Masters et al. 2000). If this hypothesis were true, one would expect to see an increasingly greater effect for the silicofluoride groups as one compared multivariable models for older dwellings with those for newer ones. Our analysis showed that, overall, the PbB concentrations of children living in counties receiving silicofluorides (sodium silicofluoride and hydrofluosilicic acid) did not differ significantly from the PbB concentrations of children living in counties without fluoridated water. When examined by year in which dwelling was built, our findings were inconsistent with our hypothesis. Among children living in dwellings of known age, silicofluorides were not associated with higher GM PbB concentrations. Specifically, with increasing dwelling age, there was no trend for an increase in the point estimates for the ratio of GM PbB concentrations, and there was no trend for an increase in adjusted odds of elevated PbB concentrations among those exposed to hydrofluosilicic acid or sodium silicofluoride, compared with no fluoride. Among children living in dwellings of unknown age, hydrofluosilicic acid was associated with a higher GM PbB concentration and an elevated PbB concentration, but sodium silicofluoride was not. Given these findings, our analysis, while not definitive, does not support concerns that silicofluorides in community water systems cause higher PbB concentrations in children.

Our investigation has several limitations. The first is the potential for exposure misclassification from use of an ecologic, county-level measure of fluoridation method. Although misclassification is always a potential threat to epidemiological studies, there is no reason to believe that misclassification in this analysis was systematic or nonrandom, and there is little reason to believe that it might have produced the observed association between silicofluorides and PbB concentrations in pre-1946 dwellings and in dwellings of unknown age. On the other hand, if a true association existed between silicofluorides and PbB concentrations, overall random misclassification could have attenuated the association.

A second limitation is the potential for confounding. We controlled at the individual level for specific risk factors for lead exposure, such as race/ethnicity, poverty status, and year in which dwelling was built. Because these variables are only proxies for actual lead exposure, we cannot exclude the possibility of residual confounding of the relation between water fluoridation method and PbB concentrations. For example, NHANES did not measure the lead content of drinking water consumed by study participants. This limitation also precluded our ability to examine more directly a potential interaction between lead in drinking water and water fluoridation method that would be expected if the hypothesized enhancement of lead uptake were correct. In addition, we did not control for community-level factors, such as density of older housing, which might be an independent risk factor for lead exposure. Finally, we were unable to control for factors that might influence the solubility of lead in pipes, including pH, temperature, and water hardness.

A third limitation is the restricted ability to reject the alternative hypothesis of relatively small but potentially important differences in PbB concentrations across water fluoridation method categories. For example, among dwellings built before 1946, the upper 95% confidence limit of the estimated GM PbB concentration ratio for hydrofluosilicic acid compared to no fluoride is consistent with a value as large as 1.7. Although no association between water fluoridation method and PbB concentrations was observed among children living in dwellings of known age, it is possible that larger samples might have identified additional, significant differences.

## Conclusions

Our analysis does not offer support for the hypothesis that silicofluorides in community water systems increase PbB concentrations in children. On the other hand, given the limitations of our data, our analyses cannot refute a possible link between water fluoridation method and lead uptake in children, particularly among those who live in older dwellings. Although other ecologic studies might allow another opportunity to test the relation between water fluoridation method and PbB concentrations in U.S. children, such analyses are likely to have similar limitations. Ultimately, the hypothesis that one or more fluoride compounds is associated with enhanced lead leaching or increased lead absorption is best addressed via systematic study of lead concentrations in drinking water, experimental chemical investigations, and studies of animal toxicology. Efforts to decrease exposure to lead among children by targeting prevention efforts at high-risk communities and/or populations as well as efforts to prevent dental caries via the use of fluoridated drinking water should continue unless a causal impact of certain fluoridation methods on PbB concentration is demonstrated by additional research.

## Figures and Tables

**Table 1:** Sample characteristics for U.S. children 1–16 years of age, by selected characteristics, 1988–1994, with estimates for the U.S. population.^a^

Characteristic	Sample size (*n* = 9,477)	Estimate^b^ (%)
Age (years)		
1–5	4,624	29.6
6–16	4,853	70.4
Sex		
Male	4,692	51.7
Female	4,785	48.3
Race/ethnicity		
Non-Hispanic white	2,551	65.1
Non-Hispanic black	3,119	15.5
Mexican American	3,338	9.2
Other	469	10.2
Poverty status		
≥ 100% FPL	5,108	70.4
< 100% FPL	3,612	24.5
Unknown	757	5.1
Urbanicity^c^		
Urban	7,373	71.9
Suburban/rural	2,104	28.1
Duration at residence		
Lifetime	3,377	31.5
Less than lifetime	3,928	49.4
Unknown	2,172	19.1
Year in which dwelling was built		
Before 1946	1,560	19.8
1946–1973	3,818	35.2
1974 to present	2,769	35.1
Unknown year	1,330	9.9
Water fluoridation method		
Unknown/mixed status	2,303	30.0
Sodium silicofluoride	1,021	10.2
Hydrofluosilicic acid	2,149	25.9
Sodium fluoride	346	7.3
Natural fluoride	1,127	8.0
No fluoride	2,531	18.6

a From the Third National Health and Nutrition Examination Survey (1988–1994) and 1992 Fluoridation Census.

b Weighted to reflect the civilian noninstitutionalized population of the United States. Persons with unknown blood lead levels were excluded from analysis.

c Urban, population ≥ 250,000; surburban/rural, population < 250,000.

**Table 2:** Weighted geometric mean (μg/dL) PbB concentrations for U.S. children 1–16 years of age, by selected characteristics, 1988–1994 (*n* = 9,477).^a^

Characteristic	GM (95% CI)^b^	Crude Wald-*F p*-value
Overall	2.19 (2.00–2.39)	—
Age (years)		< 0.01
1–5	3.09 (2.82–3.38)	
6–16	1.91 (1.74–2.09)	
Sex		< 0.01
Male	2.40 (2.19–2.63)	
Female	2.00 (1.82–2.18)	
Race/ethnicity		< 0.01
Non-Hispanic white	1.95 (1.78–2.13)	
Non-Hispanic black	3.31 (3.03–3.62)	
Mexican American	2.57 (2.35–2.81)	
Other	2.24 (1.96–2.56)	
Poverty status		< 0.01
≥ 100% FPL	1.91 (1.74–2.09)	
< 100% FPL	3.24 (2.96–3.54)	
Unknown	2.63 (2.20–3.15)	
Urbanicity^c^		0.14
Urban	2.29 (2.09–2.51)	
Suburban/rural	2.00 (1.67–2.39)	
Duration at residence		< 0.01
Lifetime	2.34 (2.14–2.57)	
Less than lifetime	2.00 (1.82–2.18)	
Unknown	2.57 (2.24–2.94)	
Year in which dwelling was built		< 0.01
Before 1946	2.95 (2.58–3.38)	
1946–1973	2.19 (2.00–2.39)	
1974 to present	1.74 (1.59–1.90)	
Unknown year	2.75 (2.41–3.15)	
Water fluoridation method		0.88
Unknown/mixed status	2.14 (1.87–2.45)	
Sodium silicofluoride	2.40 (2.00–2.87)	
Hydrofluosilicic acid	2.34 (2.05–2.68)	
Sodium fluoride	1.78^d^ (1.08–2.92)	
Natural fluoride	2.24 (1.79–2.81)	
No fluoride	2.24 (2.04–2.45)	

a From the Third National Health and Nutrition Examination Survey (1988–1994) and 1992 Fluoridation Census.

b Weighted to reflect the civilian noninstitutionalized population of the United States. Persons with unknown blood lead levels were excluded from analysis.

c Urban, population ≥ 250,000; surburban/rural, population < 250,000.

d Does not meet the standard for statistical reliability.

**Table 3:** Geometric mean PbB concentrations and ratios for U.S. children 1–16 years of age, by water fluoridation method and year in which dwelling was built, 1988–1994 (*n* = 9,477).^a^

	Before 1946			1946–1973			1974–present			Unknown		
Water fluoridation method^b^	No.	GM	Ratio^c^ (95% CI)	No.	GM	Ratio (95% CI)	No.	GM	Ratio (95% CI)	No.	GM	Ratio (95% CI)
Unknown/mixed status	473	2.57	0.93 (0.68–1.29)	837	2.04	0.93 (0.79–1.15)	674	1.66	1.02 (0.79–1.26)	319	2.57	1.07 (0.81–1.41)
Sodium silicofluoride	141	2.51	0.91 (0.63–1.32)	420	2.19	1.00 (0.76–1.32)	289	1.74	1.07 (0.85–1.35)	171	3.02	1.26 (0.95–1.66)
Hydrofluosilicic acid	448	3.55	1.29 (0.93–1.78)	839	2.09	0.95 (0.79–1.15)	605	1.86	1.15 (0.91–1.45)	257	3.48	1.45 (1.15–1.82)
Sodium fluoride	78	3.09	1.12 (0.74–1.70)	127	1.62	0.74 (0.59–0.93)	81	1.35^d^	0.83 (0.52–1.32)	60	2.09	0.87 (0.49–1.55)
Natural fluoride	113	2.40	0.87 (0.63–1.20)	419	2.63	1.20 (0.95–1.51)	413	1.70	1.05 (0.74–1.41)	182	2.40	1.00 (0.79–1.26)
No fluoride	307	2.75	Reference	1,176	2.19	Reference	707	1.62	Reference	341	2.40	Reference
Adjusted Wald-*F p*-value			0.03			0.10			0.08			0.03

a From the Third National Health and Nutrition Examination Survey (1988–1994) and 1992 Fluoridation Census.

b Weighted to reflect the civilian noninstitutionalized population of the United States; persons with unknown blood lead levels were excluded from analysis.

c Ratio of the geometric mean for each category of water fluoridation method to the geometric mean for the no-fluoride category; analysis controlled for age, sex, race/ethnicity, poverty status, urbanicity, and duration of residence.

d Does not meet the standard for statistical reliability.

**Table 4:** Prevalence and adjusted odds of an elevated PbB concentration at the 5-μg/dL cut-off for U.S. children 1–16 years of age, by water fluoridation method and year in which dwelling was built, 1988–1994 (*n* = 9,477).^a^

	Before 1946			1946–1973			1974–present			Unknown		
Water fluoridation method^b^	No.	Percent^c^	OR (95% CI)^d^	No.	Percent	OR (95% CI)	No.	Percent	OR (95% CI)	No.	Percent	OR (95% CI)
Unknown/mixed status	473	24.7	0.9 (0.4–1.9)	837	11.4	1.1 (0.4–2.7)	674	8.3	1.2 (0.5–3.2)	319	21.9	3.8 (2.0–7.0)
Sodium silicofluoride	141	20.7^e^	0.9 (0.3–2.8)	420	16.8	0.8 (0.3–2.5)	289	6.5^e^	1.0 (0.4–2.5)	171	30.1	2.8 (0.8–9.8)
Hydrofluosilicic acid	448	30.1	1.2 (0.6–2.5)	839	14.7	1.4 (0.7–2.9)	605	5.4	1.7 (0.6–4.3)	257	24.7	5.3 (2.7–10.5)
Sodium fluoride	78	20.9	0.8 (0.3–1.7)	127	7.6^e^	1.5 (0.4–5.3)	81	6.0^e^	0.6 (0.1–4.6)	60	6.6^e^	1.0 (0.3–3.6)
Natural fluoride	113	19.4	0.3 (0.1–0.6)	419	17.3	1.5 (0.7–3.2)	413	7.3^e^	1.1 (0.3–3.8)	182	16.6	1.0 (0.4–2.2)
No fluoride	307	26.4	Reference	1176	16.0	Reference	707	6.4	Reference	341	18.4	Reference
Adjusted Wald-*F p*-value			< 0.01			0.76			0.76			< 0.01

a From the Third National Health and Nutrition Examination Survey (1988–1994) and 1992 Fluoridation Census.

b Weighted to reflect the civilian noninstitutionalized population of the United States; persons with unknown blood lead levels were excluded from analysis.

c Percentage of the population with an elevated blood lead concentration (≥ 5 μg/dL).

d Adjusted OR of an elevated blood lead concentration at the 5-μg/dL cut-off, controlling for age, sex, race/ethnicity, poverty status, urbanicity, and duration of residence.

e Does not meet the standard for statistical reliability.
